# Low-Temperature Glass 3D Printing via Two-Photon and Single-Photon Polymerization of Oligo-Silsesquioxanes

**DOI:** 10.3390/polym17233204

**Published:** 2025-12-01

**Authors:** Liyuan Chen, Masaru Mukai, Yuki Hatta, Shoma Miura, Shoji Maruo

**Affiliations:** 1 Graduate School of Engineering Science, Yokohama National University, 79-5 Tokiwadai, Hodogaya-ku, Yokohama 240-8501, Japan; 2 Faculty of Engineering, Yokohama National University, 79-5 Tokiwadai, Hodogaya-ku, Yokohama 240-8501, Japan; 3 Institute of Advanced Sciences, Yokohama National University, 79-5 Tokiwadai, Hodogaya-ku, Yokohama 240-8501, Japan; 4 Institute for Multidisciplinary Sciences, Yokohama National University, 79-5 Tokiwadai, Hodogaya-ku, Yokohama 240-8501, Japan

**Keywords:** fused silica, 3D printing, two-photon polymerization, single-photon polymerization, stereolithography, sinterless resin, silsesquioxane

## Abstract

Recent advances in 3D printing of silica glass have highlighted the limitations of conventional stereolithography (SLA), which requires high-temperature sintering (≈1000 °C) and often uses slurry-based materials. To address these limitations, a sinterless approach using polyhedral oligomeric silsesquioxane (POSS)-based resin has gained attention, as it can form transparent fused silica at only 650 °C. However, previous POSS-based systems suffered from high shrinkage owing to the addition of organic monomers. In this study, a novel low-viscosity polymerizable POSS resin was synthesized without additional monomers, maintaining its sinterless properties while reducing shrinkage. Experimental results showed that our POSS resin has a silica content of 41%, with a shrinkage rate of only 36 ± 1%, which effectively reduced cracking and warping when calcinating large-volume models. It was demonstrated that this resin can be applied not only to high-resolution glass 3D printing with sub-200 nm line widths using two-photon polymerization, but also to low-cost glass 3D printing using single-photon polymerization. The 3D-printed objects can be converted into silica glass structures at significantly lower temperatures than traditional sintering, offering a promising route for efficient and precise glass manufacturing. Potential applications of our POSS resin include the production of multi-scale devices, such as microfluidic devices and optical components, and hybrid processing with semiconductors and MEMS and photonic devices.

## 1. Introduction

Glass is widely used in various fields such as optics [1], photonics [2,3], microfluidics [4,5], microelectromechanical systems (MEMS) [6], and electronics [7] because of its excellent optical transparency, thermal stability, chemical resistance, and mechanical strength. Traditional glass manufacturing methods such as blowing and mold shaping typically require extremely high temperatures to produce precise and complex structures. By contrast, the 3D printing of glass offers remarkable flexibility and precision [8,9,10,11,12], making it an attractive alternative.

Among the various 3D glass-printing techniques, stereolithography (SLA) stands out for its ultrahigh precision [13,14,15], particularly two-photon lithography, which achieves exceptional resolution [16,17,18,19]. As the raw material to produce glass 3D structures, SLA commonly uses a slurry composed of silica nanoparticles, photocurable monomers, and photoinitiators. The resulting silica/polymer composite is converted into transparent glass via debinding and sintering [13]. This method offers flexible printing of complex 3D structures, simple material preparation, and scalability to centimeter-sized objects with high resolution. Furthermore, although the fineness and roughness of the as-printed model are limited by the particle size, two-photon lithography using slurries containing nanoparticles with diameters of approximately 10 nm has been demonstrated to produce fine line structures down to sub-200 nm [16].

However, this method has certain limitations. The addition of particles can affect the refractive index of the resin, thereby causing light scattering. Therefore, matching the refractive index of the particles with that of the resin is a key challenge. The stable long-term storage of particle-dispersed slurries is also a major challenge. Furthermore, the sintering process typically requires tens of hours at temperatures above 1000 °C, reducing efficiency and increasing energy consumption. Recently, we developed a novel slurry that shortens the sintering time to 6–7 h, streamlining the production by reducing the processing time [20]. However, sintering at 1350 °C is still required to obtain transparent silica structures. This high-temperature process exceeds the melting points of commonly used resins and metals, making integration with other components and processing techniques difficult, and limiting its widespread use.

Alternatively, molecular-level resins enable 3D glass printing without the use of silica particles. Several studies [21,22,23,24] have explored photocurable organic/inorganic hybrid resins for this purpose. Notably, Bauer et al. demonstrated the fabrication of 3D microstructures with root-mean-square (RMS) roughness values less than 10 nm by two-photon lithography using cross-linkable polyhedral oligomeric silsesquioxanes (POSS) [21]. Although this POSS resin can be calcined at 600 °C for 15 h to form glass, it requires the addition of monomers to reduce its viscosity. However, the incorporation of monomers limits the silica content, resulting in approximately 42% shrinkage during calcination [21]. This significant shrinkage restricts the applicability of this material to the fabrication of microstructures via two-photon polymerization (2PP). To address this issue, Ye et al. modified the acrylate group to methacrylate and blended it with random silsesquioxane structures of different molecular weights to develop a low-viscosity POSS resin [22]. However, they applied their POSS resin to only 2PP and not single-photon SLA, which is suitable for low-cost 3D printing.

In this study, we developed a cross-linkable POSS resin with high silica content for the 3D printing of inorganic glass. Our newly developed POSS resin, designed based on its chemical composition, retained its sinterless properties while effectively suppressing shrinkage to 36 ± 1% because additional silicon-containing compounds were introduced during the synthesis process. Compared to the 42% shrinkage reported by Bauer et al. [21], our material exhibits noticeably reduced shrinkage, which helps minimize cracking and warping during calcination. Consequently, our resin enables precise 3D printing via 2PP and low-temperature calcination at 650 °C. In addition, it is adapted for single-photon SLA, which is useful in low-cost, moderately high-resolution 3D printing without expensive femtosecond lasers owing to its reduced shrinkage rate and slower shrinkage behavior. The use of two- and single-photon processes indicate the potential of the investigated system for practical glass manufacturing.

## 2. Materials and Methods

### 2.1. Material

Toluene, 2-propanol, sodium chloride, and magnesium sulfate were purchased from FUJIFILM Wako Pure Chemical Co., Ltd., Osaka, Japan. 3-(Trimethoxysilyl)propyl methacrylate (TMSPMA), tetramethylammonium hydroxide, triethoxy(isobutyl)silane, 2-(5-chloro-2-benzotriazolyl)-6-tert-butyl-p-cresol (UVA), and 2-tert-butyl-p-cresol (tBMP) were purchased from Tokyo Chemical Industry Co., Ltd., Tokyo, Japan. Diphenyl(2,4,6-trimethyl benzoyl) phosphine oxide (TPO, 97%), isobutyl (trimethoxy) silane (97%), 3-methoxy-3-methyl-1-butanol (Solifit), and chloroform-d were purchased from Sigma-Aldrich Corporation, St. Louis, MI, USA.

### 2.2. Preparation of Methacrylate-Functionalized POSS

**Figure 1:**
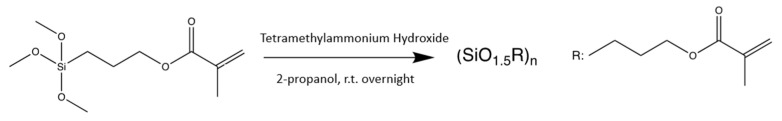
Synthesis scheme of methacrylate-functionalized POSS.

Methacrylate-functionalized POSS was synthesized using a procedure described in previous studies [21,25]. Briefly, the steps were: (i) About 120 mL of 2-propanol as the solvent and 9.4 mL of 5% tetramethylammonium hydroxide as the base catalyst were added into a reaction vessel, a magnetic stir bar was placed into the reaction vessel, and a magnetic stirrer was positioned underneath the vessel with its speed set to 780 rpm. (ii) About 9.4 g of TMSPMA was mixed evenly with 45 mL of 2-propanol. The mixture was then transferred to a dropping funnel, the drop rate was controlled, and the solution was slowly added dropwise into the previously prepared mixture in the reaction vessel. The reaction was allowed to proceed fully, and the entire process lasted approximately 24 h. During this process, the silicone compounds in the reaction vessel react and bond with each other under the influence of the base catalyst, ultimately forming a POSS structure (Figure 1). (iii) The reaction vessel was then connected to a rotary evaporator to remove the solvent. (iv) About 100 mL of toluene was then added to the reaction mixture as the solvent. After thorough mixing, the mixture was transferred to a dropping funnel. Then, saturated salt water was added to the dropping funnel, to mix with the reaction mixture. After thoroughly mixing and letting the mixture sit, the salt water was drained; fresh salt water was added, and the process was repeated three times until the reaction mixture was neutral. Then, the reaction mixture was separated. (v) Anhydrous magnesium sulfate granules were added to the modified reaction mixture for dehydration. The mixture was then filtered through a filter paper. A rotary evaporator was then used to remove the solvent from the reaction mixture, resulting in the final methacrylate-functionalized POSS of approximately 6.37 g.

^1^H NMR (400 MHz, chloroform-d): d (ppm): 6.05–5.89 (1H, Broad, CH_2_=C(CH_3_)-), 5.51–5.37 (1H, Broad, CH_2_=C(CH_3_)-), 4.11–3.92 (2H, Broad, -CH_2_CH_2_CH_2_O (C=O)-), 1.85 (3H, s, CH_2_=C(CH_3_)-), 1.76–1.50 (2H, Broad, -CH_2_CH_2_CH_2_O(C=O)-), 0.75–0.49 (2H, Broad, -CH_2_CH_2_CH_2_O(C=O)-).

### 2.3. Preparation of Cross-Linkable High-Silica Content POSS

**Figure 2:**
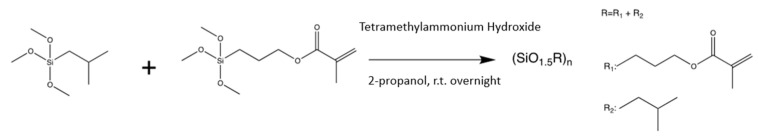
Synthesis scheme of cross-linkable high-silica content POSS.

Cross-linkable high-silica content POSS was synthesized using a method similar to that for methacrylate-functionalized POSS (Figure 2). The only difference was in step (ii) where the following operations were performed: about 4 g of TMSPMA and 3.57 g of triethoxy(isobutyl)silane were mixed evenly, then 45 mL of 2-propanol was added, and mixed together. Consequently, a composite of 4.31 g was obtained.

^1^H-NMR (400 MHz, chloroform-d): d (ppm): 6.06–5.92 (1H, Broad, CH_2_=C(CH_3_)-), 5.51–5.38 (1H, Broad, CH_2_=C(CH_3_)-), 4.11–3.92 (2H, Broad, -CH_2_CH_2_CH_2_O (C=O)-), 1.92–1.49 (6H, m, CH_2_=C(CH_3_)-, CH_2_CH_2_CH_2_O(C=O)-, -CH_2_CH(CH_3_)_2_), 0.87 (6H, s, -CH_2_CH(CH_3_)_2_), 0.70–0.38 (4H, m, -CH_2_CH_2_CH_2_O(C=O)-, -CH_2_CH(CH_3_)_2_).

### 2.4. Two Types of POSS Resins

There are two types of POSS resins: 2PP resin for micron-scale 3D structures and single-photon SLA resin for low-cost 3D printing of large-scale structures. The single-photon SLA resin is composed of four ingredients: (i) a 95 wt% cross-linkable high-silica content POSS monomer, (ii) a 1 wt% photoinitiator (TPO: diphenyl(2,4,6-trimethylbenzoyl)phosphine oxide, Sigma-Aldrich, USA), (iii) a 1 wt% photoabsorber (UVA: 2-(5-chloro-2-benzotriazolyl)-6-tert-butyl-p-cresol, Tokyo Chemical Industry Co., Ltd., Japan), and (iv) a 3 wt% polymerization inhibitor (tBMP: 2-tert-butyl-p-cresol, Tokyo Chemical Industry Co., Ltd., Japan). The 2PP resin is composed of two ingredients: (i) a 97 wt% cross-linkable high-silica content POSS monomer and (ii) a 3 wt% photoinitiator (TPO).

### 2.5. Equipment for Two-Photon Lithography

**Figure 3:**
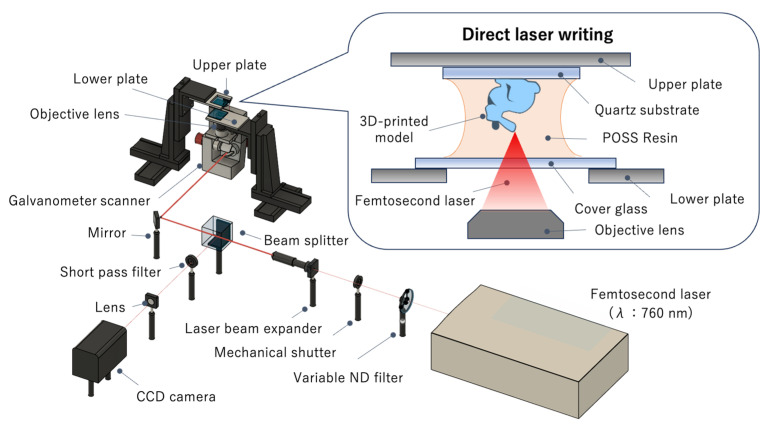
Schematic diagram of the bottom-up configuration of two-photon lithography.

Since we first demonstrated two-photon lithography in 1997 [26], we have developed various stereolithography systems, including two-photon and single-photon systems [27,28,29,30]. In this study, we used a two-photon lithography system with a femtosecond laser, with tunable oscillation wavelength (Mai Tai XF, Spectra-Physics Inc., Milpitas, CA, USA) (Figure 3). The oscillation wavelength was set to 760 nm, which is twice the wavelength of the single-photon absorption peak of the photoinitiator. The laser light was expanded 10 times using a beam expander, passed through a beam splitter, and then incident onto a galvanometer scanner (GM-1015, Canon Inc., Tokyo, Japan). The light reflected by the galvanometer mirror was focused onto the POSS resin using an objective lens (UPlanXApo, 20×/0.80, Olympus Corp., Tokyo, Japan). Within the focused volume, the photoinitiator molecules simultaneously absorb two photons and cleave to generate radicals [26]. These radicals reacted with the methacrylate groups, converting the resin into a solid polymer network consisting of an organic matrix and embedded POSS nanoclusters [21]. 3D scanning is performed by the movement of the galvanometer mirror and the XYZ-stages (OSMS20-85 (XYZ), SIGMAKOKI Co., Ltd., Saitama, Japan) supporting the upper quartz substrate (square, 20 × 20 mm), and 3D structures are formed on the quartz substrate, which has previously been irradiated with an excimer lamp (FLAT EXCIMER, EX-mini, HAMAMATSU PHOTONICS K.K., Shizuoka, Japan) to improve the adhesion of the printed 3D object.

### 2.6. Equipment for Single-Photon SLA

**Figure 4:**
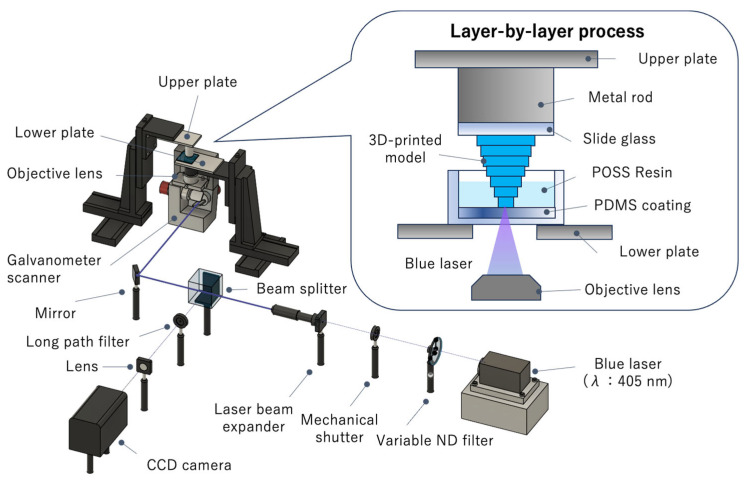
Schematic diagram of the single-photon stereolithography system.

A single-photon SLA system was also constructed in our laboratory (Figure 4) [30]. The laser light emitted from a blue semiconductor laser (Cobolt 06-MLD, Cobolt AB, Solna, Sweden; wavelength: 405 nm) was reflected using a galvanometer mirror scanner (GM-1015, Canon Inc., Tokyo, Japan). The laser light was then introduced into an objective lens (PLN4×/0.10, Olympus Corp., Tokyo, Japan) with a numerical aperture of 0.1 and focused on the POSS resin. Unlike the quartz substrate used in the 2PP system, a glass slide was used as the upper substrate and its surface was treated with methacrylic acid to ensure efficient adhesion of the 3D-printed object to the substrate during printing. A glass-bottom dish was coated with a thin film of polydimethylsiloxane (PDMS) to reduce the adhesion between the 3D-printed model and the glass substrate and facilitate peeling. A 3D structure was formed by scanning the laser beam in the x- and y- directions with galvanometer mirrors and sequentially moving in the z direction, using an XYZ stage (OSMS20-85 (XYZ), SIGMAKOKI Co., Ltd., Saitama, Japan).

### 2.7. Cleaning, Drying, and Calcination of 3D-Printed Parts

This section describes the cleaning, drying, and low-temperature calcination processes for objects 3D-printed using the 2PP and SLA methods. For 3-D printed objects using the 2PP method, 2-propanol was used to remove the excess uncured resin from the object. The cleaned 3D objects were air-dried for 24 h. POSS-based resins do not require high-temperature sintering because their printed polymerized structure already contains a densely packed silsesquioxane (Si–O) framework. After printing, moderate heating (≈650 °C) suffices to remove the organic groups and complete the condensation of the Si–O network into fused silica, thereby bypassing the need for high-temperature particle sintering [21]. In this study, the heat treatment of the 3D objects produced by 2PP was performed at 650 °C (Figure S1) in an air environment, using an electric furnace (FT-107FM-V1, FULL-TECH Co., Ltd., Osaka, Japan).

For 3D-printed objects obtained using the SLA method, 3-methoxy-3-methyl-1-butanol was used as the cleaning solution because cleaning with 2-propanol can easily damage the structure and cause small cracks. 3-Methoxy-3-methyl-1-butanol has lower permeability and volatility than 2-propanol, resulting in slower and more uniform permeation and drying. This reduced the difference between the internal and external stresses in large models, thereby reducing the occurrence of cracks. After cleaning, the 3D-printed objects were air-dried for 24 h. The heat treatment of 3D structures produced by SLA was slightly different from that used for those produced by 2PP. While both were performed in an air environment, the heat treatment of 3D structures produced by SLA was performed at 700 °C using an electric tubular furnace (KTF434N1, Koyo Thermo Systems Co., Ltd., Nara, Japan) (Figure S2). The SLA model has a larger volume than the 2PP model; therefore, even when using the same material, it is preferable to calcine at a slightly higher temperature to completely remove the organic components.

### 2.8. Characterization

The compositions of the different types of POSS resins were characterized by nuclear magnetic resonance (NMR) (EXC 400, JEOL Ltd., Tokyo, Japan). The synthesized POSS resins were polymerized and analyzed using thermogravimetric analysis (TGA) (DTG-60H, Shimadzu Corporation, Kyoto, Japan). The POSS structures were heated to 1000 °C in an air environment, and the resulting thermogravimetric profiles were used as a reference for the calcination profiles of the 3D-printed objects and for the verification of silica mass percent calculations. After 3D printing or calcination, the large-scale structures were observed and measured using an optical microscope (VHX-5000, Keyence Corporation, Osaka, Japan), and micron-scale 3D structures were observed and measured using a scanning electron microscope (SEM) (VE-8800, Keyence Corporation, Osaka, Japan). The surface roughness of the calcined models were evaluated using a laser microscope (VK-X250, Keyence Corporation, Osaka, Japan). The structures before and after calcination were characterized by energy-dispersive X-ray spectroscopy (EDX) (SU8010, Hitachi High-Tech Corporation, Tokyo, Japan). The comparison and analysis of the elemental composition of the structures before and after calcination allowed us to determine whether the structures had completed their transformation into fused silica after sintering.

## 3. Results and Discussions

### 3.1. Evaluation of Silica Content and Shrinkage Rate of Methacrylate-Functionalized POSS

In previous methacrylate-functionalized POSS research, a photocurable resin was employed, consisting of a mixture of an acrylate-functionalized POSS monomer, acrylate resin, and a photoinitiator [21]. Among these, it is believed that the acrylate resin is added to adjust the viscosity of the photocurable resin [22]. However, the addition of acrylate resin is thought to reduce the proportion of silica content and exhibited a high shrinkage (≈ 42%) after thermal treatment [21].

A key approach to minimize shrinkage is to avoid the need for added monomers or solvents to reduce the viscosity of the cross-linked POSS, while simultaneously increasing the amount of silica components in the cross-linked POSS molecule. In contrast to the acrylate-functionalized POSS used in previous studies, Ye et al. used methacrylate-functionalized POSS to build the resin [22]. They utilized a mixture of random-structure silsesquioxanes with different molecular weights to reduce the viscosity. Inspired by their approach, we hypothesized that methacrylate-functionalized POSS carries additional methyl groups that provide steric hindrance and reduce intermolecular forces, thereby lowering the overall viscosity of the substance compared to acrylate-functionalized POSS. The viscosity of methacrylate-functionalized POSS is reported to be 1.5–2.2 Pa.s at 25 °C [31], whereas the viscosity of acrylate-functionalized POSS at 25 °C is as high as 3–5 Pa.s [32]. However, while the viscosity could be reduced by using cross-linkable POSS with the acrylate group changed to a methacrylate group, the methacrylate group had one more carbon than the acrylate group; therefore, the percentage of the silica component decreased when cross-linkable POSS was used alone. To determine whether the method proposed by Ye et al. [22] is reasonable for suppressing the shrinkage rate, the calculated values of the weight loss and shrinkage rate before and after calcination, based on the composition formula, were compared with the actual measured values (Table 1).

Assuming that after calcination, all elements other than silica and the oxygen necessary to form silica disappeared, the percentage of silica content remaining after calcination in the cross-linkable POSS was estimated using Equation (1).

(1) $$Silica\;contens\;of\;POSS\left(wt\%\right)=\frac{Molecular\;weight\;of\;{SiO}_{1.5}}{Molecular\;weight\;of\;POSS\;\left(n=1\right)}\times 100$$

The calculation results showed that the silica content of acrylate-functionalized POSS was 36 wt%, whereas that of methacrylate-functionalized POSS was 33 wt%. When using POSS alone as photocurable resin, the acrylate-functionalized POSS has an advantage in terms of silica content. However, previous studies used a mixture of acrylate-functionalized POSS (rate of POSS resin = 0.89) and photocurable resin, which reduced the total silica content of the resin to 32 wt%, as shown in Equation (2).

(2) $$Total\;silica\;contens\;of\;resin\;\left(wt\%\right)=Rate\;of\;POSS\;in\;resin\times Silica\;contens\;of\;POSS$$

If methacrylate-functionalized POSS could be fabricated into a 3D structure by SLA, it would be slightly superior in terms of the silica content, as calculated from the structural formula. This silica content is thought to correspond to the weight loss associated with calcination, and the weight loss associated with calcination was measured by TGA and reported in previous papers by both Bauer et al. [21] and Ye et al. [22]. It has been reported that the residual weight remaining by TGA measurement of the POSS used by Bauer et al. [21] was 35 wt%, whereas the residual weight remaining of the POSS used by Ye et al. [22] was 41.9 wt%. Regarding the weight loss expected from the chemical composition, the results obtained by Bauer et al. were relatively close to the calculated values, whereas those obtained by Ye et al. showed an error of nearly 10 wt%. Ye et al. prepared another resin from POSS (POSS_2 in Table 1), which they eliminated because it would eventually self-polymerize spontaneously and therefore, would be impractical [22]. For POSS_2, the weight loss was compared between the values calculated based on the chemical composition and the actual values measured by TGA. The calculated and measured values for POSS_2 were 40 and 46 wt%, respectively. Although the difference was smaller than that for the methacrylate-functionalized POSS, there was a large gap between the measured and calculated values. Additionally, the shrinkage rate was calculated from density and weight loss due to heating, as shown in Equation (3).

(3) $$Caluclated\;shrinkage=\sqrt[{3}]{Total\;silica\;contens\;of\;resin\;\left(wt\%\right)/100\times Density\;of\;silica}/\sqrt[{3}]{Density\;of\;resin}\times 100$$

The density of silica was set to 2.2 g/cm^3^ based on the density of quartz [33]. The densities of the resins were 1.23 g/cm^3^ for the acrylate-functionalized POSS by Bauer et al. [34], 1.113 g/cm^3^ for the mixed resin [35], 1.2 g/cm^3^ for the methacrylate-functionalized POSS by Ye et al. [36], and 1.2 g/cm^3^ for POSS_2 (assumed to be the same as that for the methacrylate-functionalized POSS, since no value was found in the literature). Similar to the weight loss results, the shrinkage rate for POSS by Bauer et al. [21] was calculated as 45% and the actual measured value was 42%, which were close to each other; whereas for POSS by Ye et al. [22], the shrinkage rate showed a large discrepancy between the actual and calculated values.

### 3.2. Synthesis of Methacrylate-Functionalized POSS and Evaluation of Shrinkage Rate

**Figure 5:**
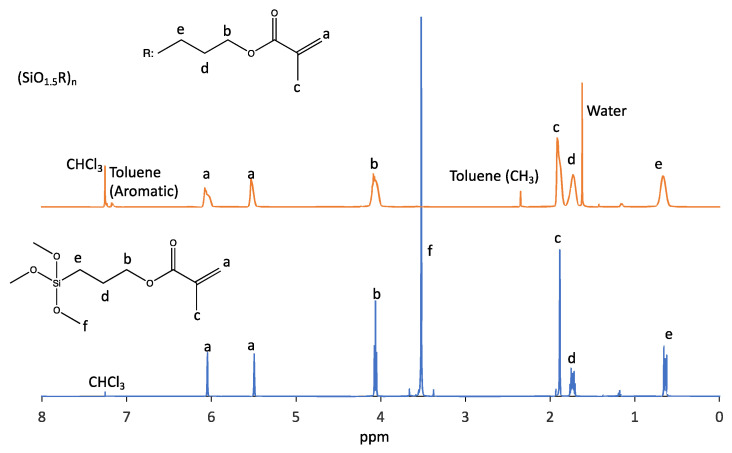
^1^H-NMR spectra of methacrylate-functionalized POSS and its precursor as 3-(trimethoxysilyl)propyl methacrylate.

Methacrylate-functionalized POSS was synthesized to confirm the results reported by Ye et al. [22]. Consequently, a liquid compound was obtained. ^1^H-NMR and ^29^Si-NMR confirmed the synthesis. The disappearance of the ^1^H-NMR peak at 3.52 ppm due to the methoxy group of the raw material suggested that the methoxy group was hydrolyzed (Figure 5, peak f). Silanol groups generated by the hydrolysis of methoxysilyl groups are usually unstable, and it is assumed that these silanol groups react with each other to form siloxane bonds [37,38]. The broadening of the NMR peaks also suggested the formation of siloxane bonds. When trimethoxysilyl groups, such as those in the raw material, randomly form siloxane bonds, a network structure is formed, resulting in a gel with no fluidity. However, because the synthesized compound was a liquid, it was difficult to consider it as having a network structure. In the reactions carried out in this study under dilute conditions, intramolecular reactions were more prevalent than intermolecular reactions, and cage-shaped silsesquioxanes were more likely to be formed [39]. Mass spectrometry (Figure S3) also revealed peaks derived from oligo POSS corresponding to T5, T6, T7, T8, and T9. This material was confirmed to be composed of a mixture of multiple POSS compounds, similar to the POSS synthesized by Ye et al. [22]. In ^29^Si-NMR, multiple peaks were observed in the −66 to −69 ppm range (Figure S4), similar to the mass spectrometry results, suggesting that the compound was a mixture of multiple POSS compounds.

**Figure 6:**
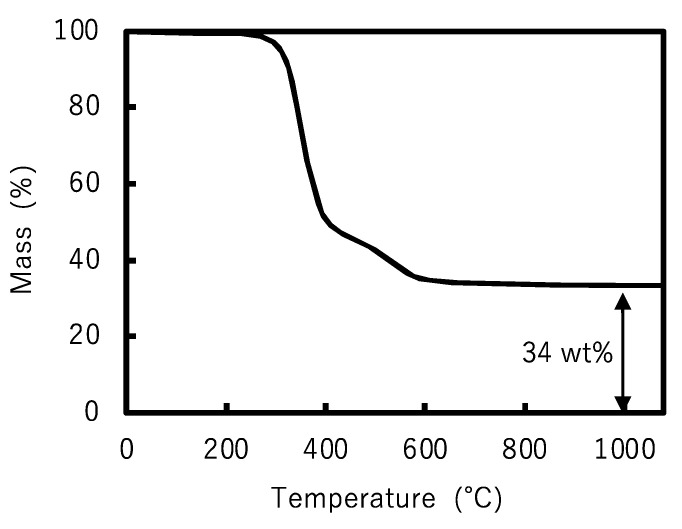
Thermogravimetric analysis curve of methacrylate-functionalized POSS resin.

A photocurable POSS resin was then prepared by adding 95 wt% synthesized POSS, 1 wt% TPO, 1 wt% UVA, and 3 wt% tBMP. Then, 5 × 5 × 0.5 mm plates were fabricated by the SLA method using this photocurable resin. The plates were then subjected to TGA. TGA measurements showed that the percentage of residual components above 1000 °C was 34% (Figure 6). This weight loss was attributed to the organic component of the photocurable resin that was decomposed and removed by heating, and the remaining main component was presumed to be silica. The weight of the residue was in good agreement with the 33 wt% (Table 2) proportion of the silica component obtained from Equation (1), but not with the TGA results of Ye et al. of 41.9 wt% [22]. In addition, the TGA curve (Figure 6) also shows a rapid weight loss at around 400 °C. This result is consistent with prior literature [25] and suggests that calcination at low temperatures below 1000 °C is possible.

Once we were able to print a 3D object using the SLA method, we attempted to form a glass structure by calcination; however, cracks appeared after calcination, and we were unable to obtain a transparent calcined body because the objects produced by the SLA method were larger than those produced by the 2PP method. In larger models, uneven heat transfer during the heating and cooling phases of calcination can result in varying shrinkage rates in different areas. This uneven shrinkage generates internal stress, resulting in the warping and cracking of the model. This is particularly true in models with larger wall thicknesses. As the heating and cooling rates used in the study (Figure S2) were already low, further optimization of the heat transfer uniformity during the calcination process was inefficient. Therefore, reducing the overall shrinkage rate can be an effective strategy.

To confirm the shrinkage rate, we performed 3D printing using 2PP with methacrylate-functionalized POSS and investigated the shrinkage rate of the 3D-printed object after calcination (Table S1 and Figure S5). Three samples were measured before and after calcination using SEM, and the average values were obtained from these measurements. In this case, the methacrylate-functionalized POSS could be calcined without breaking, and the shrinkage rate was 44 ± 1% (Table 2). Because the shrinkage rate and weight loss were similar to the calculated values, our experiment showed a higher shrinkage rate than that reported by Ye et al. [22]. However, in a previous report by Bauer et al. [21], the calculated values were almost the same as the experimental values; therefore, our evaluation method is considered valid. Hence, we assume that the shrinkage rate and weight loss reported by Ye et al. [22] were underestimated owing to their evaluation method or the presence of impurities in the resin.

### 3.3. Evaluation of Silica Content and 3D Printing of Cross-Linkable High-Silica Content POSS

**Figure 7:**
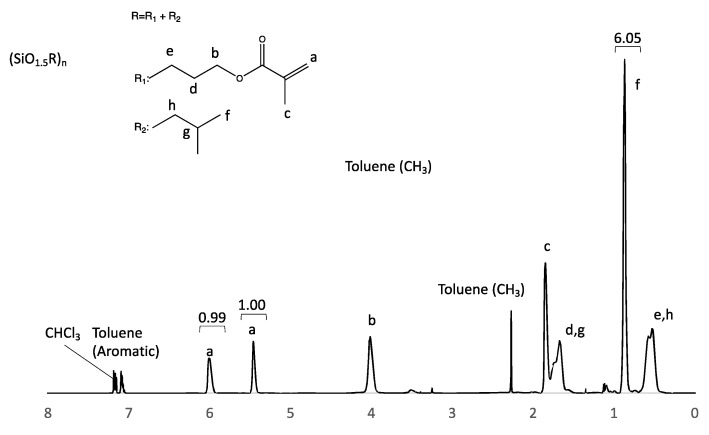
^1^H-NMR spectra of cross-linkable high-silica content POSS.

**Figure 8:**
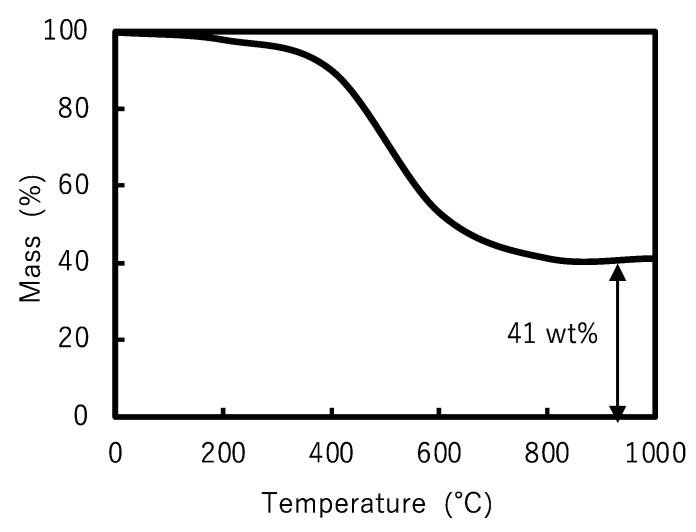
Thermogravimetric analysis curve of cross-linkable high-silica content POSS resin.

From the aforementioned results, it is assumed that SLA-compatible resins require a higher silica content. Therefore, the organic functional groups of POSS that are effective in increasing the silica content were investigated. The decrease in the organic components, including carbon, increased the relative proportion of silica components. For example, the numbers of carbon atoms in the propyl methacrylate, propyl acrylate, and isobutyl groups were 6, 5, and 4, respectively. If all eight organic functional groups of POSS were isobutyl groups, the silica content would be the highest. However, octa-isobutyl-substituted POSS is a solid [40]. It has no polymerizable functional groups; therefore, it loses its curability and crosslinking ability. We investigated formulations that are liquid at room temperature and maintain their crosslinking properties. To increase the silica content, a cross-linkable POSS was synthesized, and half of the eight propyl methacrylate groups comprising the methacrylate-functionalized POSS were replaced with isobutyl groups. The final synthesized POSS monomer is a low-viscosity liquid that can be used directly without adding any monomers, and the silica content can reach 41% in the chemical structure from Equation (1). The synthesis was carried out in a manner similar to that for methacrylate POSS, and ^1^H-NMR and ^29^Si-NMR confirmed the synthesis. In this methacrylate-functionalized POSS, the peaks derived from the methoxysilyl group disappeared (Figure 7), and the POSS was liquid. ^29^Si-NMR suggested that cage-like silsesquioxanes were synthesized. When the integral value of 5.51–5.38 ppm assigned to one of the methacrylate-group terminal hydrogen atoms was taken as 1, the integral value of 6.06–5.92 ppm assigned to another methacrylate-group terminal hydrogen atom was 0.99, and the integral value of 0.87 ppm assigned to the isobutyl-group terminal hydrogen atom was 6.05 (Figure 7). Multiple peaks in the −65 to −69 ppm range of ^29^Si-NMR suggest that this liquid is a mixture of multiple POSS (Figure S6). The hydrogen atom number of the isobutyl group at the end was six, which is nearly consistent with the integral value obtained by NMR, suggesting that this compound is formed by an almost 1:1 reaction between a silane coupling agent with a methacrylate group and a silane coupling agent with an isobutyl group. This result is consistent with the ratio of the raw materials used in the synthesis. As with methacrylate POSS, the actual silica content was estimated from TGA using SLA and was found to be 41% (Figure 8), which agreed with the calculated value (Table 2). Compared to the cross-linkable POSS used in previous studies [21], mixed with light-curing resin, our cross-linkable high-silica-content POSS greatly improved the silica content.

**Figure 9:**
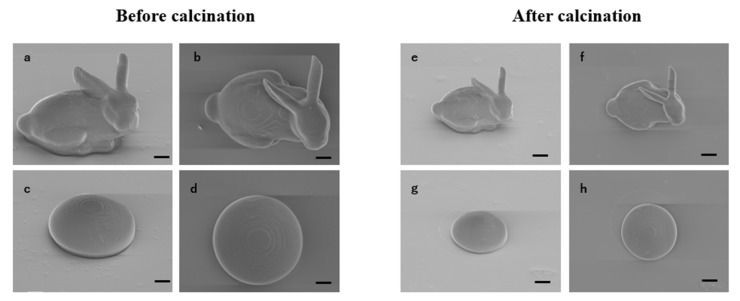
SEM images of fabricated bunny and microlens models before and after calcination. (**a**,**b**) Bunny models before calcination. Scale bar: 20 μm. (**c**,**d**) Microlens models before calcination. Scale bar: 20 μm. (**e**,**f**) Bunny models after calcination. Scale bar: 20 μm. (**g**,**h**) Microlens models after calcination. Scale bar: 20 μm.

After confirming the increase in the proportion of silica, the resin was first used for 2PP 3D-printing, and models of a bunny and microlens were printed (Figure 9). During the printing process, a laser power of 150 mW was applied, with the layer thickness set to 1 μm, and a total height of 150 layers for the bunny and 100 layers for the microlens. The SEM images revealed 3D structures with smooth surfaces, showing no noticeable stair-stepping effects or cracks. The fine details of the bunny model (Figure 9a,b), such as the ears, were well-preserved without distortion, demonstrating the excellent performance of the resin in high-complexity geometric shaping. The microlens (Figure 9c,d) exhibited uniform layering and good surface quality, indicating stable adhesion between the layers during the curing process. After calcination, the model retained its semi-spherical shape with no noticeable collapse, cracks, or uneven shrinkage (Figure 9e,h).

The shrinkage of 3D-printed models fabricated with cross-linkable high-silica-content POSS resins using 2PP was evaluated after calcination (Table S2 and Figure S7). Three samples were measured before and after calcination using SEM, and the average values were obtained from these measurements (Table 2). The results showed a reduced isotropic linear shrinkage of 36 ± 1%, which is smaller than the 42% isotropic shrinkage reported by Bauer et al. [21]. This confirms that the synthesized crosslinked, high-silica-content POSS resin had a higher silica content than the acrylate-functionalized POSS resin. The results of previous studies by Bauer et al. [21] and Ye et al. [22] as well as the data for the two materials synthesized in this study are summarized in Table S4.

**Figure 10:**
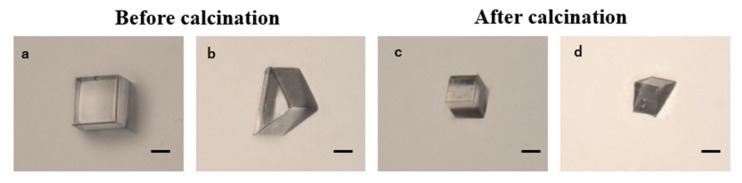
Optical images of fabricated cube and prism models before and after calcination. (**a**,**b**) Cube and prism models before calcination. Scale bar: 20 μm. (**c**,**d**) Cube and prism models after calcination. Scale bar: 20 μm.

To achieve smoother structures, we fabricated a cube and a triangular prism with a layer thickness of 50 nm. By observing the calcined models, we confirmed the formation of highly transparent microstructures (Figure 10). Using a laser microscope (VK-X250, Keyence Corporation, Osaka, Japan), we measured the surface roughness of the top surface of the cube and the side surface of the triangular prism. The surface roughness of the top surface of the cube (a circular area with a diameter of 20 μm) and the side surface of the triangular prism correspond to the surface roughness in the xy plane and the z direction (the stacking direction) of the 3D printing, respectively. Regarding the surface roughness in the stacking direction, the prism models were laid out on the substrate after calcination (Figure 10d)) such that the surface roughness of the side surfaces in the stacking direction could be measured from above. The surface roughness Sa of the top surface of the cube was 3 nm, and the surface roughness, Ra, of the side surface of the triangular prism was 77 nm. These results indicate that while excellent in-plane smoothness of several nanometers was achieved, the surface roughness in the stacking direction was on the order of tens of nanometers, depending on the layer pitch. According to the Maréchal criterion [41], which defines the acceptable surface roughness for optical components as approximately λ/14 of the operating wavelength, the in-plane surface roughness of this sample (3 nm) is well below this threshold and is fully suitable for optical applications. The sidewall roughness (77 nm) is significant because of the 2PP stacking structure and is influenced not only by the material properties, but also by fabrication conditions such as layer pitch, scanning parameters, and exposure settings. Although the layer pitch in this experiment was 50 nm, it is possible that the surface roughness could be reduced by further reducing the layer pitch. Furthermore, even greater precision may be achieved by using grayscale lithography, which dynamically adjusts the voxel size by controlling the exposure dose [42].

**Figure 11:**
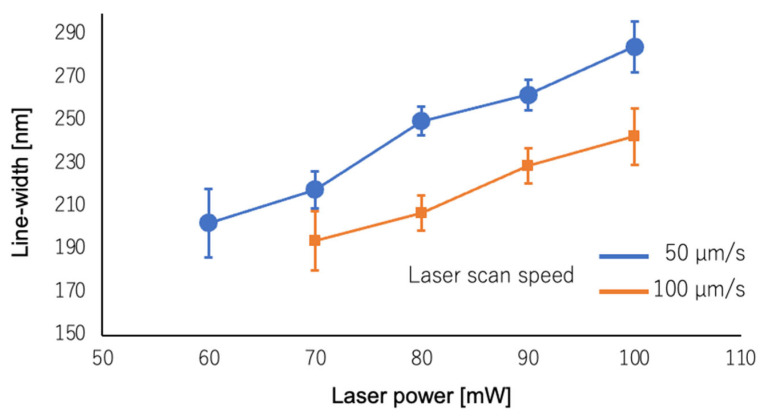
Dependence of the line-width on laser power and laser scanning speed.

Next, the fabrication resolution of the cross-linkable high-silica-content POSS resin was evaluated. Single-line structures were fabricated on a quartz substrate using an objective lens (UPlanSApo, 100×/1.4, Olympus Corp., Tokyo, Japan), and the line width was measured after calcination. The laser-scanning speed was set to 50 and 100 μm/s, and the laser power was varied between 30 and 120 mW. The curing line widths were measured using SEM (Figure 11). Consequently, a faster laser scanning speed resulted in a smaller processed line-width, whereas a lower laser power resulted in a smaller descending line-width. A minimum line-width of approximately 194 nm was observed (Figure S8). The resolution achieved in this study was slightly lower than the minimum linewidths of 119 nm and 75 nm reported by Bauer et al. [21] and Ye et al. [22], respectively. As our POSS resin has a composition similar to that used by Ye et al. [22], we believe that the inherent precision, which depends on the resin, is comparable. However, the resolution is highly dependent not only on the resin formulation, but also on the manufacturing equipment, printing parameters, and cleaning process. Therefore, we believe that the difference in the minimum linewidth observed here is due to the differences in the printing system and cleaning process. In the future, we plan to further improve the printing parameters and cleaning process to increase the achievable feature precision.

**Figure 12:**
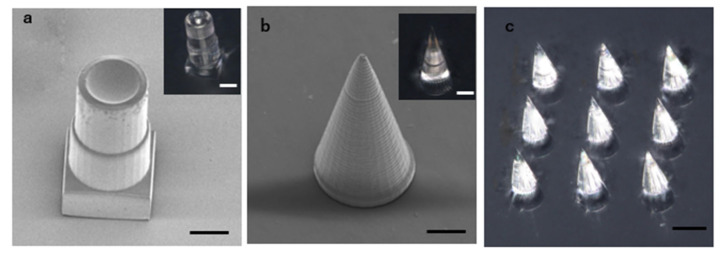
Silica glass models made by single-photon SLA method. (**a**) Nozzle model after calcination. Scale bar: 50 μm. (**b**) Needle model after calcination. Scale bar: 50 μm. (**c**) Needle array after calcination. Scale bar: 100 μm.

The cross-linkable high-silica-content POSS resin not only facilitates precise 3D printing through 2PP but is also suitable for the low-cost 3D printing of large-scale models using a bottom-up approach based on the single-photon SLA method. We fabricated nozzle and needle models (Figure 12a,b) with a laser power of 5 mW, layer thickness of 10 μm, and a total height of 25 layers. SEM observations of the calcined models showed that the structures maintained good fidelity with no significant deformation or collapse. Additionally, observations using optical microscopy (VHX-5000) confirmed their transparency after calcination. The shrinkage rates (Table S3) of the single-photon SLA model (Figure S9) was generally consistent with the 2PP results.

**Figure 13:**
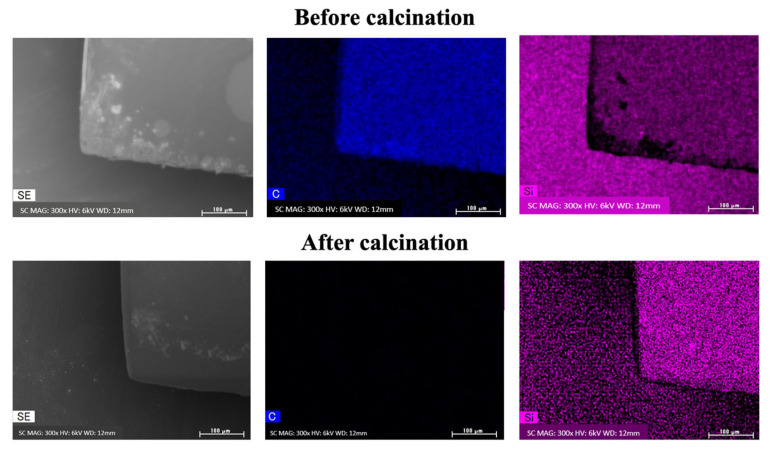
EDX results of the green and calcined bodies. Scale bar: 100 μm.

To confirm the composition of the 3D models obtained from the previous experiments, we conducted EDX experiments of the 3D-printed models, before and after calcination. Before calcination, carbon components derived from the organic matter were detected. However, after calcination, these carbon components disappeared, and the silicon components remained (Figure 13). The TGA results showed that the weight remained essentially unchanged after 650 °C, and the transmittance data further confirmed that the calcined samples exhibited high transparency consistent with glass materials (Figure S11). Thus, it can be concluded that the resin has been fully converted into a 3D glass structure at this point.

## 4. Conclusions

We developed a cross-linkable high-silica content POSS resin for the free-form manufacturing of silica glass; our POSS resin retained the advantage of enabling the conversion to fused silica at only around 650 °C, similarly to that reported in a previous study [21]. As an improvement, we replaced acrylate with methacrylate to reduce the viscosity of POSS, and added triethoxy(isobutyl)silane to increase its silica content. Experimental results showed that our POSS resin has a silica content of 41%, with a shrinkage rate of 36 ± 1%. Compared with the 42 ± 1% shrinkage rate in previous Bauer et al. study [21], our shrinkage rate was lower, which effectively reduced cracking and warping when calcinating large-volume models. Additionally, the contraction rate of the synthesized POSS was in good agreement with that predicted from its chemical structure and was structurally reasonable. Our newly developed, cross-linkable, high-silica-content POSS resin not only enables high-resolution 3D printing through 2PP, but is also applicable for low-cost, single-photon SLA with moderate resolution. Depending on the required feature size and 3D model size, using either 2PP or single-photon SLA can expand the flexibility and applicability of glass 3D printing. For example, the adaptability of this resin allows flexible size adjustments in the production of multiscale devices, including microfluidic devices [43,44] and optical components [45], while maintaining high precision from the micron scale to macroscopic dimensions. Our POSS resin can also be applied to single-photon polymerization using an ultraviolet lamp, potentially enabling the fabrication of microfluidic devices by encapsulating a 3D-printed mold in the POSS resin, followed by exposure and calcination to remove the 3D-printed mold [46,47,48]. Our POSS resin not only significantly lowers the conversion temperature of fused silica to below the melting points of many common materials, but also provides multi-scale manufacturing capabilities, making it promising for hybrid processing with semiconductors and MEMS and photonic devices.

## Figures and Tables

**Table 1:** Calculated values of shrinkage and weight loss for different POSS types, and their actual values in prior literature by Bauer et al. [21] and Ye et al. [22].

	Composition Formula of POSS	Silica Contents of POSS	Total Silica Contents of Resin	Remaining Weight Percentage of TGA	Calculated Shrinkage	Observed Shrinkage
Bauer et al. acrylate-functionalized POSS	(SiO_1.5_(C_6_H_9_O_2_))_n_	36 wt%	32 wt%	35 wt%	45%	42 ± 1%
Ye et al. methacrylate-functionalized POSS	(SiO_1.5_(C_7_H_11_O_2_))_n_	33 wt%	33 wt%	41.9 wt%	43%	33%
Ye et al. POSS_2	(SiO_1.5_(C_5_H_7_O_2_))_n_	40 wt%	40 wt%	46.2 wt%	39.9%	28.5%

**Table 2:** Calculated values of shrinkage and weight loss and measured values in our synthesized methacrylate-functionalized POSS and cross-linkable high-silica-content POSS.

	Composition Formula of POSS	Silica Contents of POSS	Total Silica Contents of Resin	Remaining Weight Percentage of TGA	Calculated Shrinkage	Observed Shrinkage
Our synthesized methacrylate-functionalized POSS resin	(SiO_1.5_(C_7_H_11_O_2_))_n_	33 wt%	33 wt%	34 wt%	43%	44 ± 1%
Cross-linkable high-silica content POSS resin	(SiO_1.5_(C_7_H_11_O_2_)_0.5_(C_4_H_9_)_0.5_)_n_	42 wt%	42 wt%	41 wt%	37%	36 ± 1%

## Data Availability

The datasets generated and/or analyzed in the current study are available from the corresponding author upon reasonable request.

## Supplementary Materials

The following supplementary material can be downloaded at https://www.mdpi.com/article/10.3390/polym17233204/s1, Figure S1: Temperature-time profile of 3D structures printed by 2PP with methacrylate-functionalized POSS resin and cross-linkable high-silica content POSS resin; Figure S2: Temperature-time profile of 3D structures printed by single-photon SLA with methacrylate-functionalized POSS resin and cross-linkable high-silica content POSS resin; Figure S3: Mass spectrometry results of methacrylate-functionalized POSS resin; Figure S4: ^29^Si-NMR spectra of methacrylate-functionalized POSS and its precursor as 3-(trimethoxysilyl)propyl methacrylate; Figure S5: 2PP-printed cylindrical models made from methacrylate-functionalized POSS resin for shrinkage evaluation. (a) 3D-printed resin model before calcination. (b) Calcinated silica model. (c) Diameter measurement of cylindrical models. (d) Height measurement of cylindrical models; Figure S6: ^29^Si-NMR spectra of cross-linkable high-silica content POSS; Figure S7: 2PP-printed cylindrical models made from cross-linkable high-silica content POSS resin for shrinkage evaluation. (a) 3D-printed resin model before calcination. (b) Calcined silica model. (c) Diameter measurement of cylindrical models. (d) Height measurement of cylindrical models; Figure S8: SEM image of a single-line structure with a minimum line width of 194 nm; Figure S9: Single-photon SLA models made from cross-linkable high-silica content POSS resin, used for measuring the shrinkage rate. Scale bar: 200 μm; Figure S10: Transmittance of a flat plate (4 mm × 4 mm × 100 μm) fabricated using single-photon SLA with cross-linkable high-silica-content POSS resin before calcination; Figure S11: Transmittance of a flat plate (4 mm × 4 mm × 100 μm) fabricated using single-photon SLA with cross-linkable high-silica-content POSS resin after calcination; Table S1: Analysis of the dimensions and shrinkage rates (in all directions) of the 2PP model before and after calcination, which was made from methacrylate-functionalized POSS resin; Table S2: Analysis of the dimensions and shrinkage rates (in all directions) of the 2PP model before and after calcination, which was made from the cross-linkable high-silica content POSS resin; Table S3: Analysis of the dimensions and shrinkage rates (in all directions) of the single-photon SLA model before and after calcination, which was made from the cross-linkable high-silica content POSS resin; Table S4: Comparison of shrinkage and weight loss for all POSS types.

## Abbreviations

The following abbreviations are used in this manuscript:

SLA	stereolithography
POSS	polyhedral oligomeric silsesquioxane
2PP	two-photon polymerization
MEMS	microelectromechanical systems
RMS	root mean square
UVA	2-(5-chioro-2-benzotriazolyl)-6-tert-butyl-p-cresol
tBMP	2-tert-butyl-p-cresol
TPO	diphenyl(2,4,6-trimethyl benzoyl) phosphine oxide
TMSPMA	3-(trimethoxysilyl)propyl methacrylate
PDMS	polydimethylsiloxane
NMR	nuclear magnetic resonance
TGA	thermogravimetric analysis
SEM	scanning electron microscope
EDX	energy-dispersive X-ray spectroscopy
