# Supplementary material for: Low-Temperature Glass 3D Printing via Two-Photon and Single-Photon Polymerization of Oligo-Silsesquioxanes
Source: Polymers (Basel). 2025 Dec 1;17(23):3204. doi: 10.3390/polym17233204 (PMC12694004; doi:10.3390/polym17233204)
Supplement: Supplementary file 1 [file polymers-17-03204-s001.zip › polymers-3967823-supplementary.pdf]

Supplementary Material

Low-Temperature Glass 3D Printing via Two-Photon and Single-Photon Polymerization of Oligo-Silsesquioxanes

Liyuan Chen, Masaru Mukai, Yuki Hatta, Shoma Miura, and Shoji Maruo

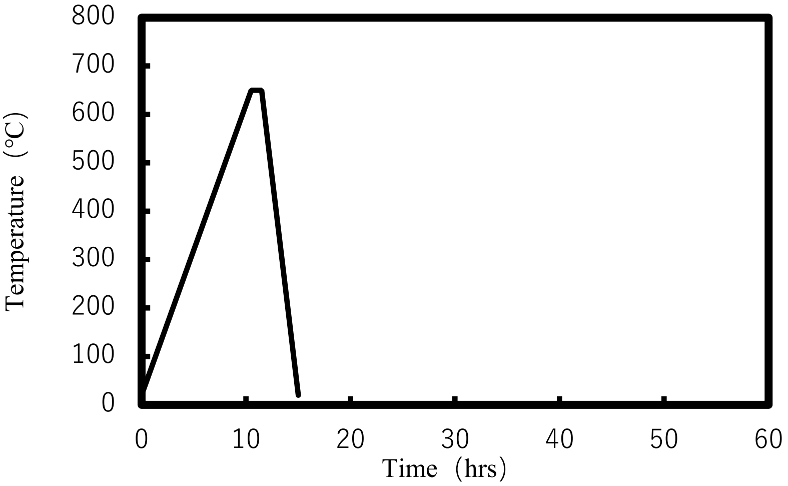

Figure S1. Temperature-time profile of 3D structures printed by 2PP with methacrylate-functionalized POSS resin and cross-linkable high-silica content POSS resin.

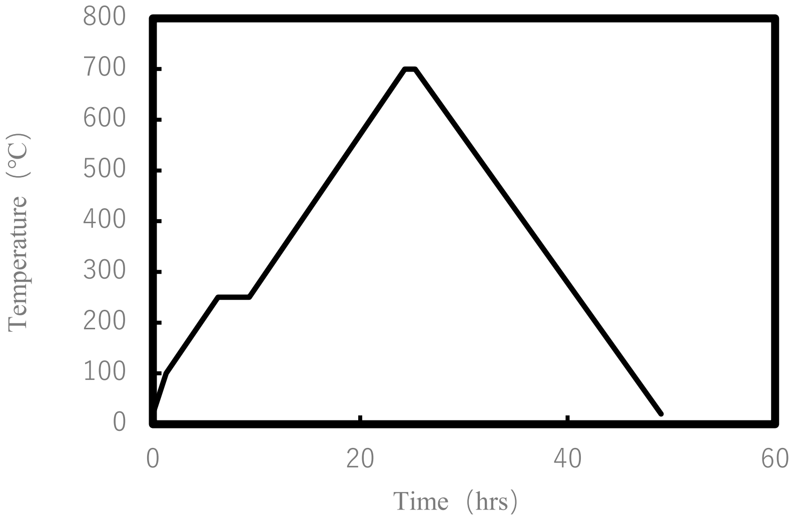

Figure S2. Temperature-time profile of 3D structures printed by single-photon SLA with methacrylate-functionalized POSS resin and cross-linkable high-silica content POSS resin.

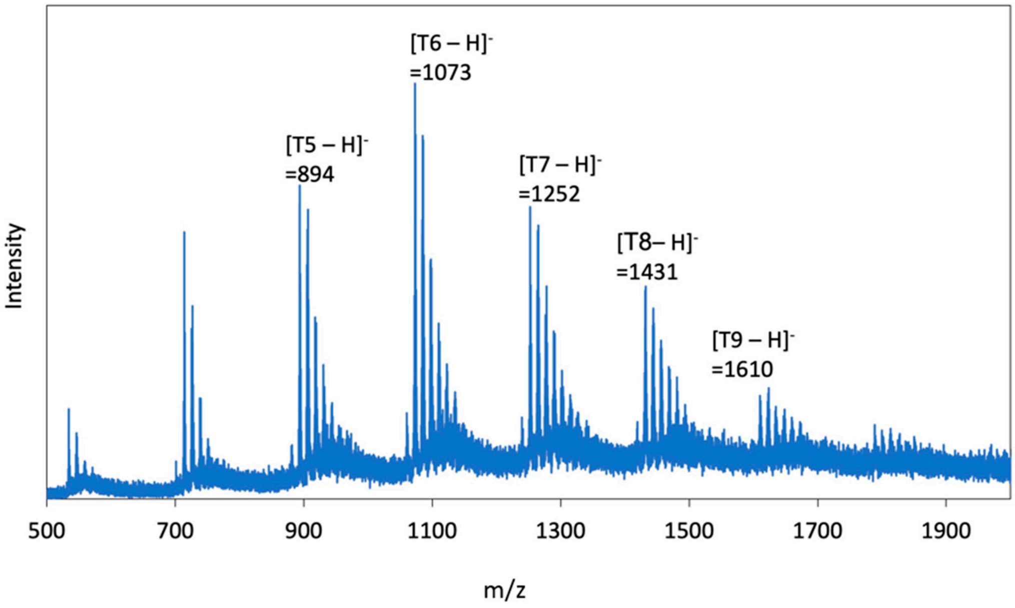

Figure S3. Mass spectrometry results of methacrylate-functionalized POSS resin.

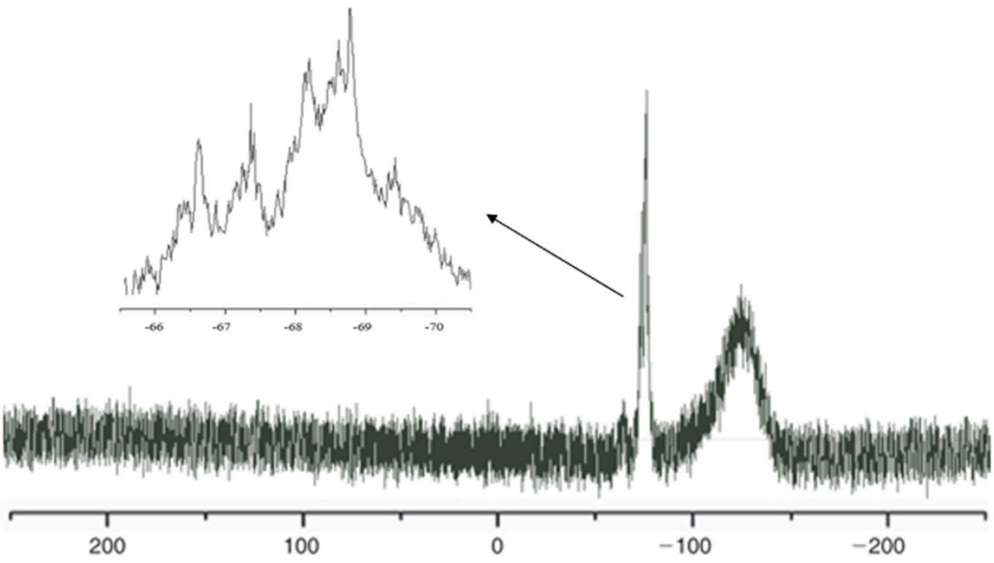

Figure S4. ^{29}Si-NMR spectra of methacrylate-functionalized POSS and its precursor as 3-(trimethoxysilyl)propyl methacrylate. (The broad peak around −100 ppm originates from the NMR tube.)

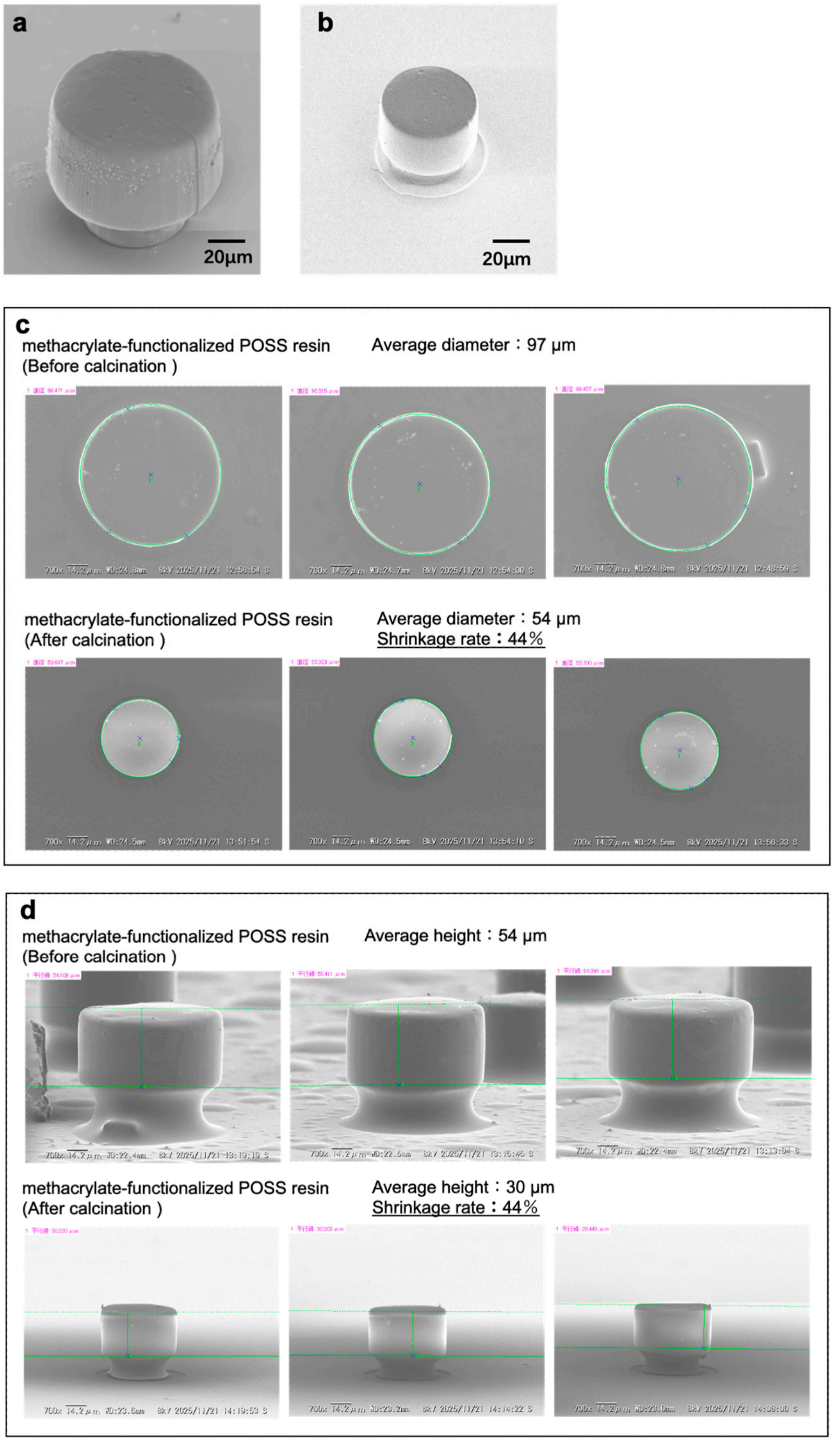

Figure S5. 2PP-printed cylindrical models made from methacrylate-functionalized POSS resin for shrinkage evaluation. (a) 3D-printed resin model before calcination. (b) Calcinated silica model. (c) Diameter measurement of cylindrical models (d) Height measurement of cylindrical models.

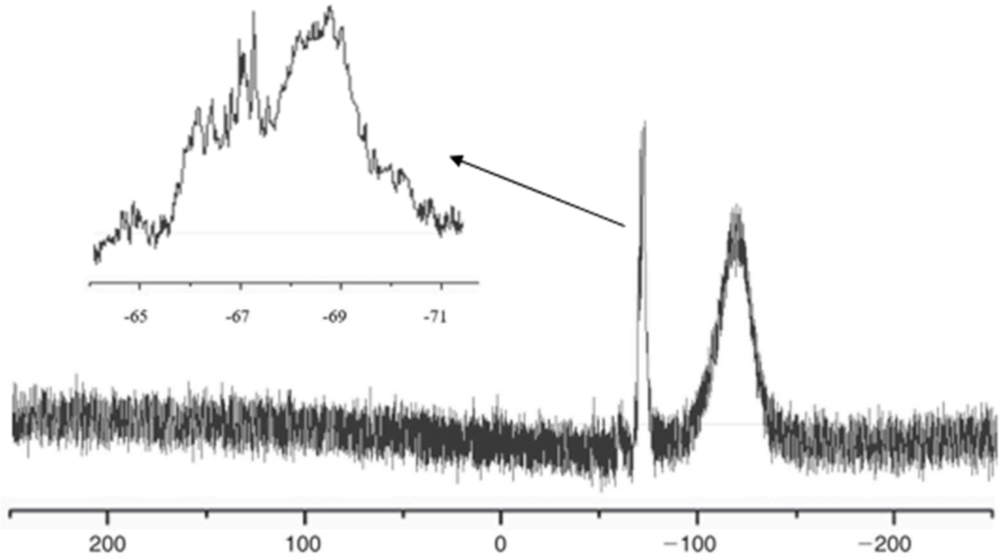

Figure S6. ^{29}Si-NMR spectra of cross-linkable high-silica content POSS. (The broad peak around −100 ppm originates from the NMR tube.)

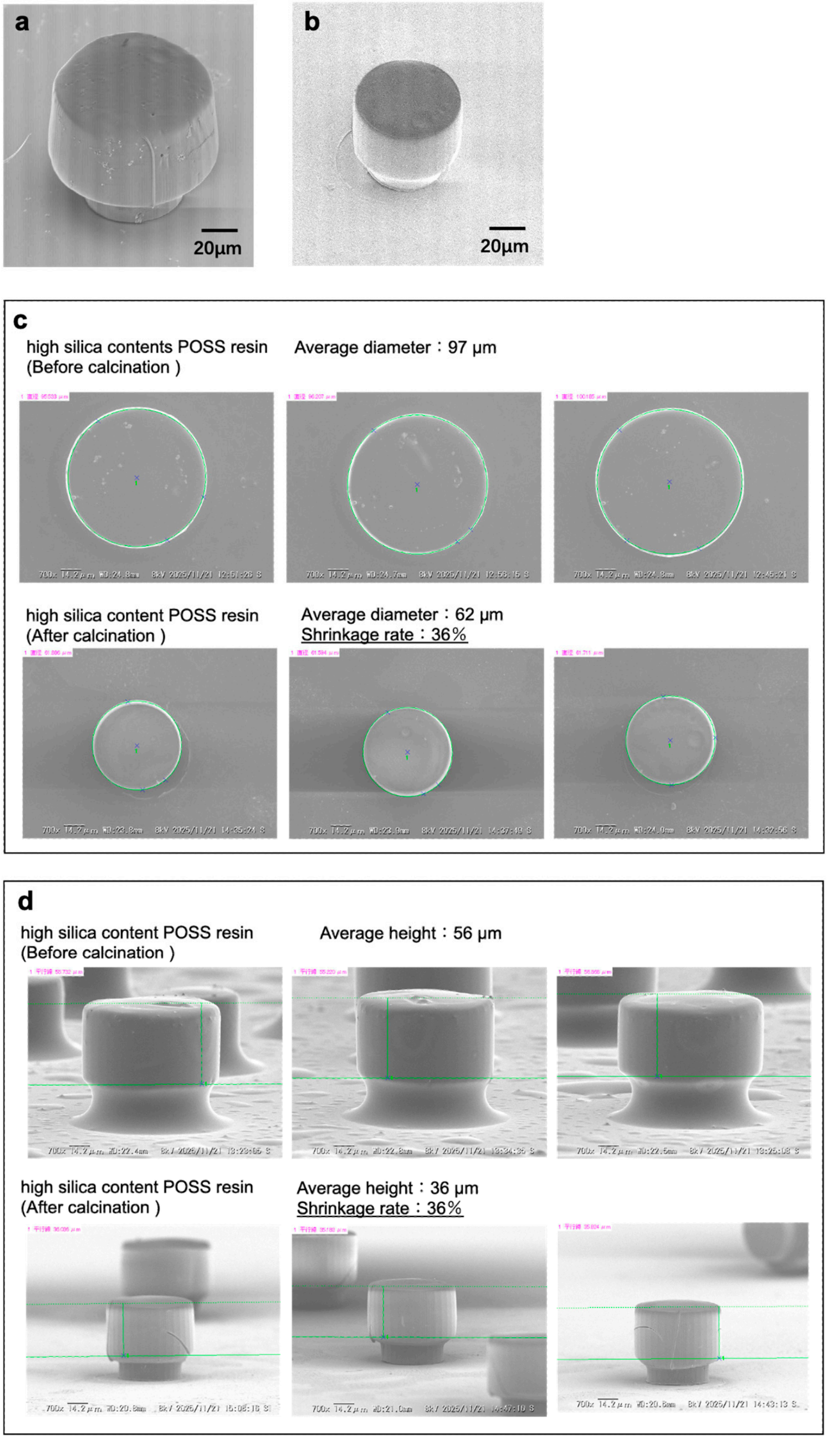

Figure S7. 2PP-printed cylindrical models made from cross-linkable high-silica content POSS resin for shrinkage evaluation. (a) 3D-printed resin model before calcination. (b) Calcined silica model. (c) Diameter measurement of cylindrical models (d) Height measurement of cylindrical models.

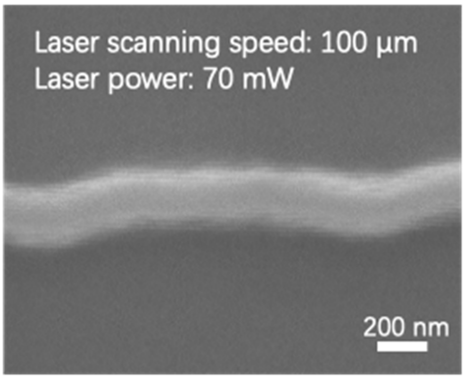

Figure S8. SEM image of a single-line structure with a minimum line width of 194 nm.

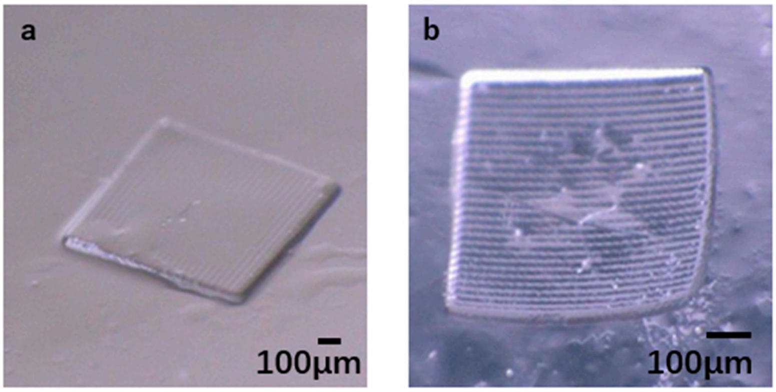

Figure S9. Single-photon SLA models made from cross-linkable high-silica content POSS resin, used for measuring the shrinkage rate. Scale bar: 200 μm.

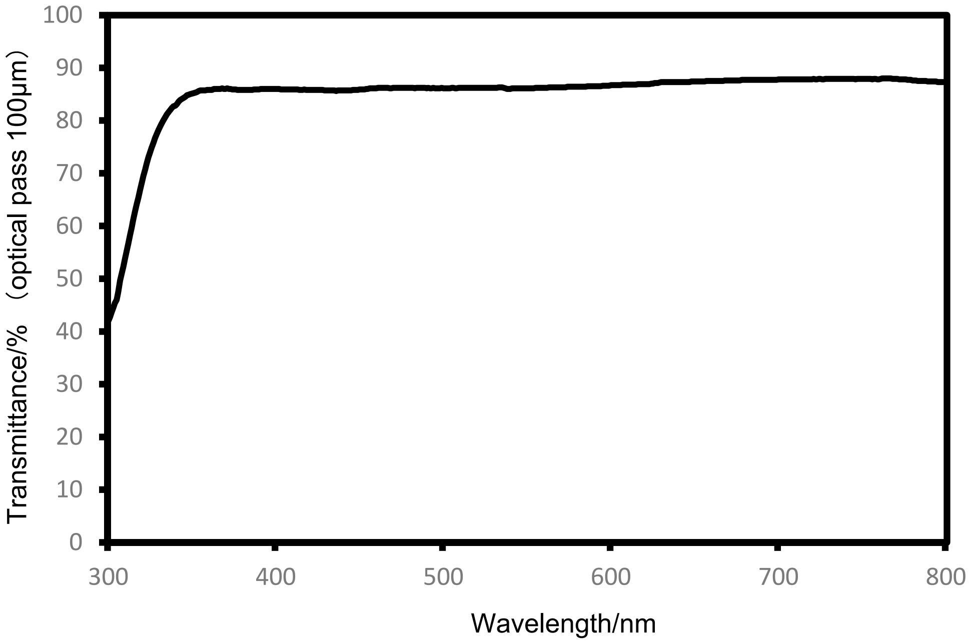

Figure S10. Transmittance of a flat plate (4 mm × 4 mm × 100 μm) made by single-photon SLA with cross-linkable high-silica content POSS resin before calcination.

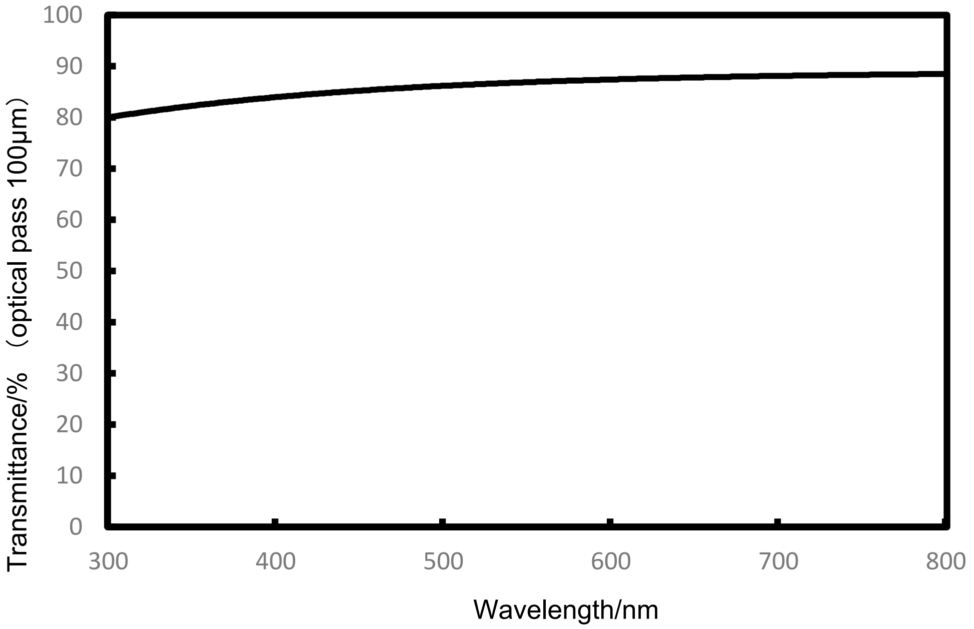

Figure S11. Transmittance of a flat plate (4 mm × 4 mm × 100 μm) made by single-photon SLA with cross-linkable high-silica content POSS resin after calcination.

Table S1. Analysis of the dimensions and shrinkage rates (in all directions) of the 2PP model before and after calcination, which was made from methacrylate-functionalized POSS resin.

2PP model (Cylinder)	3D-printed model (μm)	Calcined model (μm)	Shrinkage rate (%)
Diameter	97	54	44±1
Hight	54	30	44±1

Table S2. Analysis of the dimensions and shrinkage rates (in all directions) of the 2PP model before and after calcination, which was made from the cross-linkable high-silica content POSS resin.

2PP model (Cylinder)	3D-printed model (μm)	Calcined model (μm)	Shrinkage rate (%)
Diameter	97	62	36±1
Hight	56	36	36±1

Table S3. Analysis of the dimensions and shrinkage rates (in all directions) of the single-photon SLA model before and after calcination, which was made from the cross-linkable high-silica content POSS resin.

Single-photon model (Thin plate)	3D-printed model (μm)	Calcined model (μm)	Shrinkage rate (%)
Length	836	528	37±1
Hight	1000	648	35±1
Thickness	78	51	35±1

Table S4. Comparison of shrinkage and weight loss for all POSS types

	Composition formula of POSS	Silica contents of POSS	Total silica contents of resin	Remaining weight percentage of TGA	Calculated Shrinkage	Observed Shrinkage
Bauer et al. acrylate-functionalized POSS	$(SiO_{1.5}(C_6H_9O_2))_n$	36 wt%	32 wt%	35 wt%	45%	42±1%
Ye et al. methacrylate-functionalized POSS	$(SiO_{1.5}(C_7H_{11}O_2))_n$	33 wt%	33 wt%	41.9 wt%	43%	33%
Ye et al. POSS_2	$(SiO_{1.5}(C_5H_7O_2))_n$	40 wt%	40 wt%	46.2 wt%	39.9%	28.5%
Our synthesized methacrylate-functionalized POSS	$(SiO_{1.5}(C_7H_{11}O_2))_n$	33 wt%	33 wt%	34 wt%	43%	44±1%
Cross-linkable high-silica content POSS	$(SiO_{1.5}(C_7H_{11}O_2)_{0.5}(C_4H_9)_{0.5})_n$	42 wt%	42 wt%	41 wt%	37%	36±1%
